# CD14^+^-Monocytes Exposed to Apolipoprotein CIII Express Tissue Factor

**DOI:** 10.3390/ijms24032223

**Published:** 2023-01-22

**Authors:** Oliviero Olivieri, Sara Gasperini, Federica Calzetti, Elisa Gardiman, Annalisa Castagna, Nicola Martinelli, Nicola Tamassia, Marco A. Cassatella

**Affiliations:** 1 Unit of Internal Medicine, Department of Medicine, University of Verona, 37134 Verona, Italy; 2 Section of General Pathology, Department of Medicine, University of Verona, 37134 Verona, Italy

**Keywords:** apolipoprotein CIII, tissue factor, monocytes, thrombosis, atherosclerosis

## Abstract

Apolipoprotein CIII (ApoCIII) represents a key regulator of plasma lipid metabolism and a recognized risk factor for atherosclerosis and cardiovascular diseases. Beyond the regulation of lipoprotein trafficking, ApoCIII is also involved in endothelial dysfunction and monocyte recruitment related to atherothrombosis. With tissue factor (TF) being the primary initiator of the blood coagulation cascade, we hypothesized that ApoCIII-treated monocytes could express it. Hence, human CD14^+^-monocytes and autologous neutrophils were incubated with ApoCIII and sera from human subjects containing previously measured ApoCIII amounts. By RT-qPCR and ELISA, CD14^+^-monocytes, but not neutrophils, were found to show increased mRNA expression and production of TNFα, IL-1β and IL-6 as well as TF mRNA once exposed to ultra-purified ApoCIII. By flow cytometry, CD14^+^-monocytes were found to rapidly express TF on their cell surface membrane when incubated with either ApoCIII or sera with known concentrations of ApoCIII. Finally, preincubation with specific ApoCIII-neutralizing antibodies significantly reduced the ability of most sera with known concentrations of ApoCIII to upregulate TF protein, other than partially inhibiting cytokine release, in CD14^+^-monocytes. In sum, herein we demonstrate that ApoCIII activates CD14^+^-monocytes to express TF. The data identify a potential mechanism which links circulating apolipoproteins with inflammation and atherothrombosis-related processes underlying cardiovascular risk.

## 1. Introduction

Apolipoprotein CIII (ApoCIII) is a key regulator of plasma lipid metabolism, especially of triglyceride (TG)-rich lipoproteins (TRLs). ApoCIII is also recognized as an important risk factor for ischemic heart disease [1,2] and for the development of atherosclerotic disease [3]. Although such an atherogenic role is commonly attributed to altered lipid mechanisms—in particular, to the inhibition of lipoprotein lipase (LPL)-mediated lipolysis of chylomicrons and very low-density lipoproteins (VLDLs), with consequent TG-rich remnants’ accumulation—other mechanisms have been also demonstrated. For instance, some of them involve direct effects of the apoprotein on endothelial cells or more generally on the inflammatory aspects of the arteriosclerotic process [4,5,6]. The development of atheroma requires, in addition to the lipoprotein accumulation, concomitant inflammation of the arterial wall. ApoCIII was found to be involved in the early steps of this process, including in endothelial dysfunction and in the recruitment of blood monocytes to the site of injury and their subsequent leaking into the subendothelial space [4,5,6].

More recently, Speer and colleagues [7] provided novel experimental evidence for alternative inflammasome activation in human monocytes exposed to ApoCIII (i.e., via a signaling pathway involving TLR-2 and TLR-4 dimerization and then caspase-8 activation). In fact, after incubation with different species of lipoproteins (i.e., high-density lipoproteins (HDLs), low-density lipoproteins (LDLs) and VLDLs), they found that only VLDLs stimulate the secretion of IL-1β by monocytes. Then, by using delipidated VLDL, they revealed that only the protein component of VLDL was the factor responsible for NLRP3 inflammasome activation. More specifically, this effect was found to be mediated by ApoCIII, which was also able to induce the release of tumor necrosis factor-α (TNFα) and interleukin 6 (IL-6). ApoCIII was also found to trigger a calcium influx and increased superoxide production by monocytes [7]. Altogether, the intriguing observations made by Speer and colleagues [7] highlight a central role of ApoCIII in the inflammation that occurs in vessels affected by an active atherosclerotic process. They also indirectly suggest that plaque lesions rich in ApoCIII may trigger and promote the coagulation cascade involving monocytes, ultimately leading to thrombotic (and generally dramatic) complications of atherosclerotic disease. Monocytes are in fact able to express tissue factor (TF) under pro-inflammatory stimuli, including C-reactive protein, TNFα, LPS, CD40 and angiotensin II [8]. In addition, it has also been indicated that VLDLs (but not other types of lipoproteins) can increase the expression of FVII and plasminogen activator inhibitor I (PAI-1) in endothelial cells [9] and monocytes [10]. Consistent with these experimental findings, previous clinical results also suggest a “procoagulant” activity of ApoCIII in the setting of both arterial atherosclerotic vascular disease and venous thromboembolism [11,12,13,14]. 

Therefore, considering that TF is the primary initiator of the blood coagulation cascade, we hypothesized that monocytes could express TF on their surface when “fired up” by ApoCIII [15]. The aim of the present study was therefore to verify such a hypothesis by investigating whether monocytes express TF upon exposure to ApoCIII. Answering this question would help to better understand the multifaceted and harmful role of ApoCIII in the pathogenesis of atherothrombotic disease. 

## 2. Results

**Figure 1:**
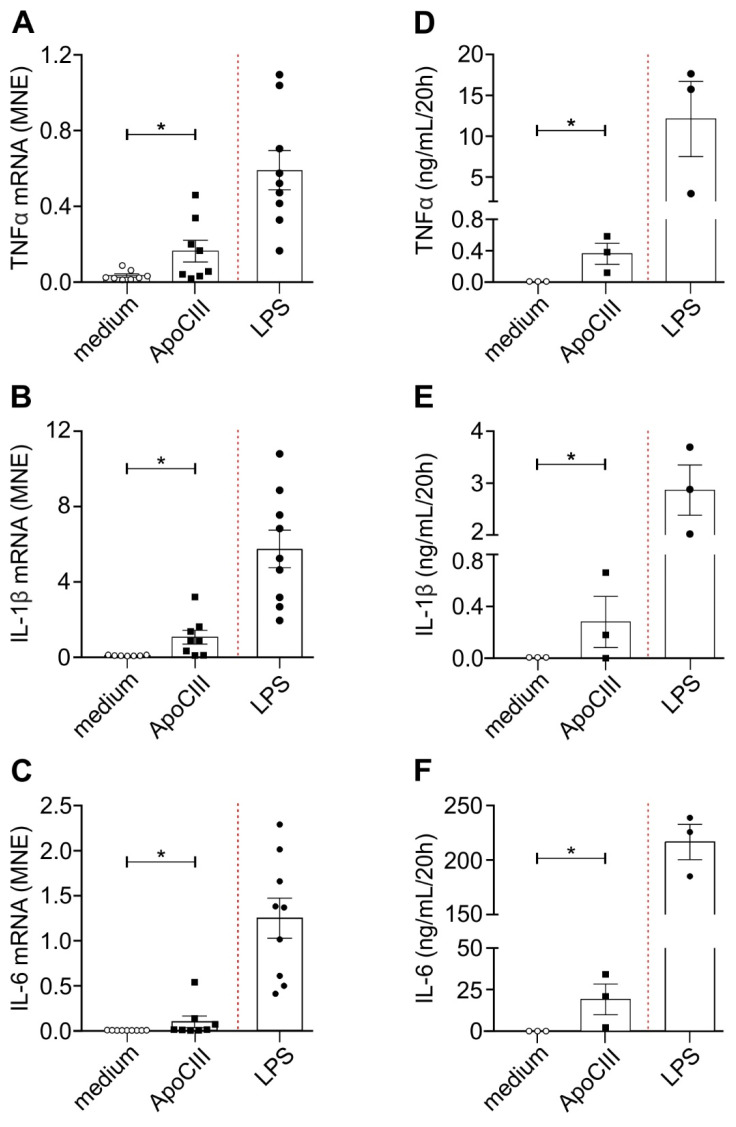
Expression of TNFα, IL-1β and IL-6 mRNAs and protein release by human CD14^+^-monocytes exposed to ApoCIII. Human CD14^+^-monocytes, purified as described in Section 4, were incubated for 3 h (**A**–**C**) or 20 h (**D**–**F**) in the absence (medium) or presence of 50 µg/mL ultrapure ApoCIII or, as a control, in 100 ng/mL LPS. Total RNA was then extracted and examined for TNFα (**A**), IL-1β (**B**) and IL-6 (**C**) mRNA expression by RT-qPCR. Gene expression is depicted as mean normalized expression (MNE) units after normalization to GAPDH mRNA (mean ± SEM, *n* = 7–9). Monocyte-derived supernatants were collected, and TNFα (**D**), IL-1β (**E**) and IL-6 (**F**) levels were measured by ELISA. Results are expressed as the mean value ± SEM of 3 independent experiments. Statistical analysis by unpaired t-test with Welch correction refers to ApoCIII-treated versus untreated monocytes, * *p* < 0.05.

Initial experiments confirmed [7] that incubation with 50 µg/mL ultrapure ApoCIII (commercially purchased and purified as described in Section 4) triggers significant levels of TNFα (Figure 1A), IL-1β (Figure 1B) and IL-6 (Figure 1C) mRNA expression in CD14^+^-monocytes.

Such ApoCIII-mediated effects on cytokine mRNA expression were found to be more evident after 3 rather than 20 h of incubation (Supplementary Figure S1A–C), but less potent than those exerted by either 100 ng/mL LPS (Figure 1) or 5 µM R848 (also known as Resiquimod) (Supplementary Figure S1A–C). ApoCIII was found to trigger TNFα (Supplementary Figure S1D) and IL-1β (Supplementary Figure S1E) but not IL-6 (Supplementary Figure S1F) mRNA expression also in autologous neutrophils, but at lower levels than in CD14^+^-monocytes and, as expected, than with LPS or R848. As shown in Figure 1, the patterns of cytokine mRNA induction (Figure 1A–C) substantially matched the production of TNFα, IL-1β and IL-6 by CD14^+^-monocytes measured after 20 h of cell incubation (Figure 1D–F). Notably, all ApoCIII-mediated effects on cytokine gene expression by both monocytes (Supplementary Figure S2) and neutrophils were insensitive to the preincubation of ApoCIII with polymyxin B (PMX). The data prove that the endotoxin contaminating the commercially purchased ApoCIII was successfully eliminated by the (*ad hoc* used) endotoxin removal kit, and that, in turn, the observed ApoCIII-mediated effects on phagocytes were ascribable to ApoCIII.

**Figure 2:**
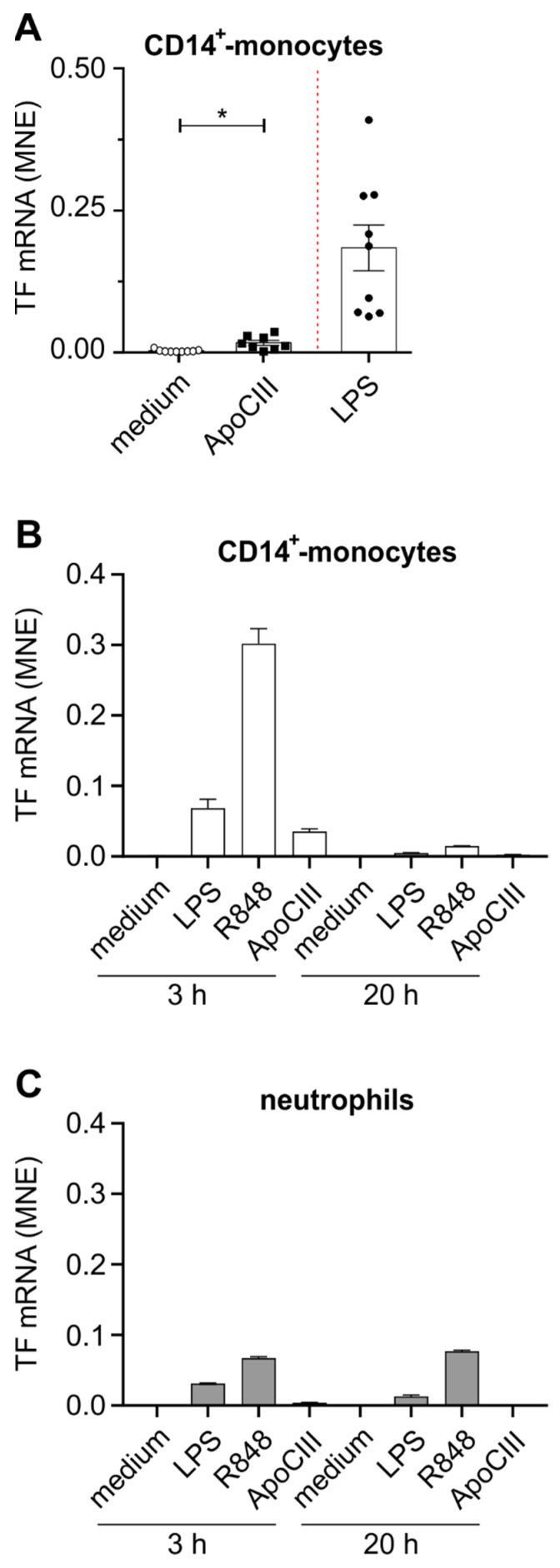
Expression of tissue factor (TF) mRNA in human CD14^+^-monocytes and autologous neutrophils exposed to ApoCIII. Human CD14^+^-monocytes (**A**,**B**) and autologous neutrophils (**C**), purified as described in Section 4.2, were incubated for 3 h (**A**–**C**) and/or 20 h (**B**,**C**) in the absence (medium) or presence of 50 µg/mL ApoCIII, 100 ng/mL LPS (**A**,**B**), 1 µg/mL LPS (**C**) or 5 µM R848 (**B**,**C**). Total RNA was then extracted and examined for TF mRNA expression by quantitative RT-qPCR. Gene expression is depicted as MNE units after normalization to GAPDH mRNA. For panel **A**, data are represented as mean ± SEM, *n* = 8–9, and were analyzed by unpaired t-test with Welch correction. * *p* < 0.05. Panels **B** and **C** show a representative experiment from a single donor (out of 2 performed with similar results). Data are represented as mean ± SEM of experimental replicates.

When investigating TF mRNA expression in these same experiments, we found a significant TF mRNA accumulation in ApoCIII-treated CD14^+^-monocytes (Figure 2A,B) but not in autologous neutrophils (Figure 2C). 

**Figure 3:**
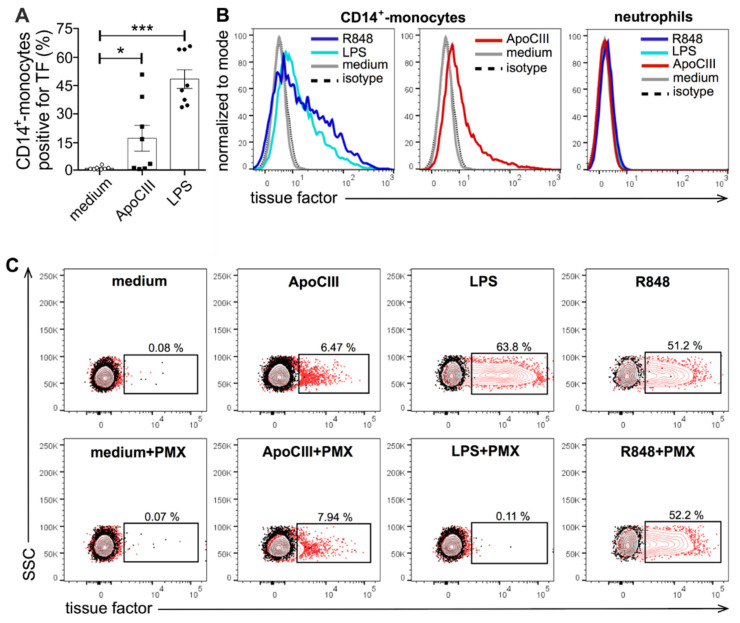
Expression of surface TF in human CD14^+^-monocytes and autologous neutrophils exposed to ApoCIII. Human CD14^+^-monocytes (**A**–**C**) and autologous neutrophils (**B**), purified as described in Section 4, were incubated for 3 h in the absence (medium) or presence of 50 µg/mL ApoCIII, 5 µΜ R848 or 0.1 and 1 µg/mL LPS for CD14^+^-monocytes and neutrophils, respectively. In selected experiments, the stimuli were preincubated with 20 µg/mL polymyxin B (PMX) before their addition to the cells (**C**). Cells were then stained for surface TF expression by FACS as described in Section 4. Panel **A** depicts the percentage of TF^+^ CD14^+^-monocytes (mean ± SEM, n = 8). Data were analyzed by one-way ANOVA followed by the Holm–Sidak post hoc test. * *p* < 0.05; *** *p* < 0.001. Panel **B** displays representative histograms showing the expression of surface TF by CD14^+^-monocytes and neutrophils incubated with ApoCIII, LPS and R848 as compared to isotype control antibodies. Panel **C** displays representative flow cytometry plots showing the percentage of TF^+^ CD14^+^-monocytes (represented as red dots) incubated with ApoCIII, LPS and R848, in the absence or presence of PMX, as compared to the isotype control antibodies (represented as black dots).

However, TF mRNA expression could be induced by both LPS and R848 in the latter cells (Figure 2C) as well as in CD14^+^-monocytes, doing so in both cell types at higher levels than those promoted by ApoCIII. As for cytokine genes, TF mRNA accumulation in ApoCIII-treated CD14^+^-monocytes was also more elevated after 3 rather than 20 h of incubation (Figure 2B) and insensitive to PMX (Figure S2D). Notably, monocytes, but not neutrophils, treated with ApoCIII for 3 h were found to display surface TF by flow cytometry (Figure 3A,B), with such an effect being much less pronounced after 20 h, and again, PMX-insensitive (Figure 3C), thus substantiating the gene expression data. Nonetheless, both LPS and R848 were found to promote the expression of surface TF in CD14^+^-monocytes more potently than ApoCIII (Figure 3A,B), with LPS, but not R848, being (as expected) neutralized by PMX (Figure 3C). 

Other leukocyte types such as slan^+^-monocytes and B and T lymphocytes were found not to express surface TF under the same experimental conditions. Notably, by ELISA, we also did not detect soluble TF in cell-free supernatants harvested from either ApoCIII- or LPS/R848-treated CD14^+^-monocytes. The latter data are consistent with the notion that TF is a transmembrane protein which needs to be relocated in the phospholipid bilayer to bind and efficiently activate FVII [8]. 

**Figure 4:**
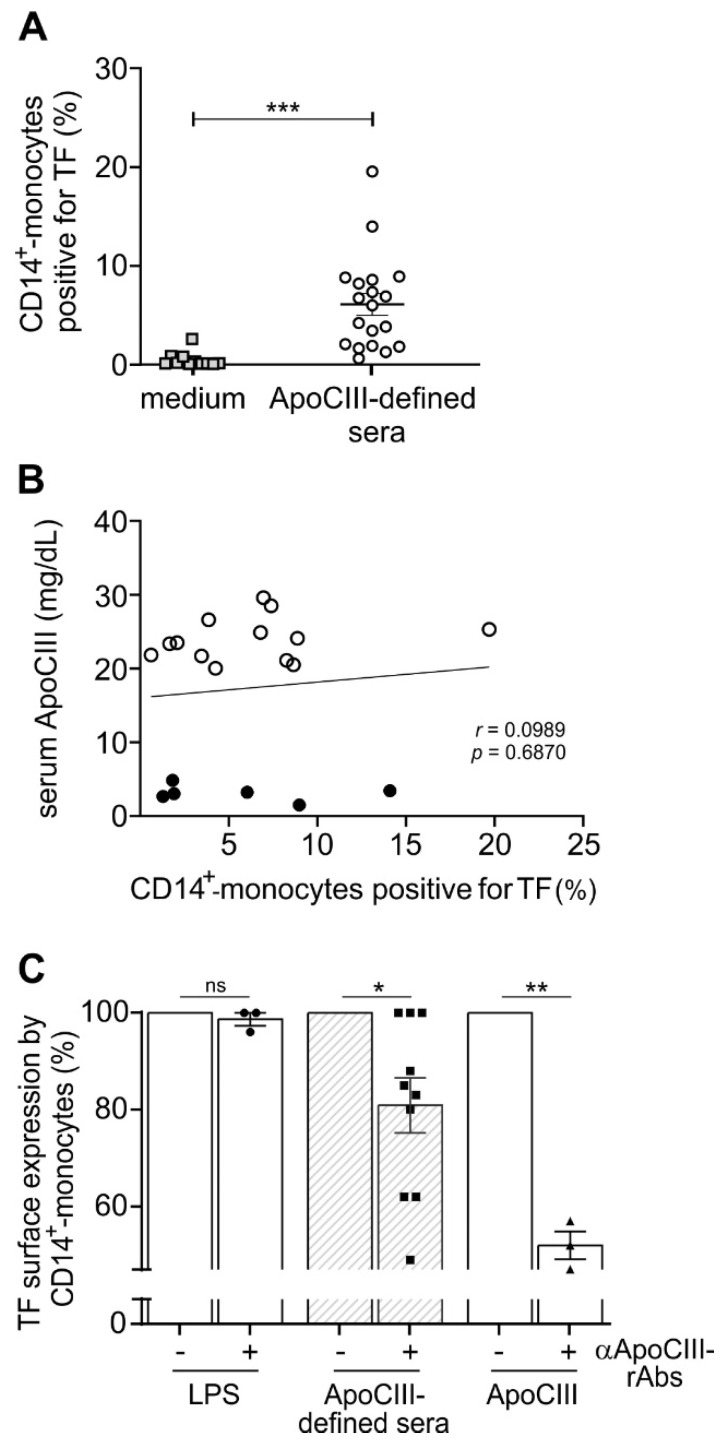
Effect of sera with measured concentrations of ApoCIII on surface TF expression by CD14^+^-monocytes. Panel **A** shows the percentages of CD14^+^-monocytes expressing TF from PBMCs incubated for 3 h with or without 10% sera obtained from 19 individuals displaying different serum ApoCIII levels ranging from 1.5 to 29.6 mg/dL. Data are expressed as mean ± SEM (*n* = 11–19) and were analyzed by unpaired *t*-test with Welch correction. *** *p* < 0.001. Panel **B** displays a scatter plot with a linear regression curve showing the relationship between the percentages of CD14^+^-monocytes expressing TF and the ApoCIII concentrations of the sera used to stimulate PBMCs. The r-value determined by Pearson correlation is displayed. Panel **C** reports CD14^+^-monocytes expressing TF from PBMC samples incubated for 3 h with either 50 µg/mL ApoCIII, sera with known concentrations of ApoCIII (ApoCIII-sera) or 100 ng/mL LPS in the presence or absence (as 100%) of 60 µg/mL ApoCIII-neutralizing rAbs. Statistics of data refer to the effects of anti-ApoCIII-recombinant antibodies (αApoCIII-rAbs) on each treatment, and were evaluated by one sample t-test. * *p* < 0.05; ** *p* < 0.01; *n* = 3–10.

In a subsequent series of experiments, PBMCs were incubated for 3 h with 10 % sera obtained from 19 individuals with ApoCIII concentrations ranging from 1.5 to 29.6 mg/dL (which is equivalent to the distribution range of ApoCIII that is normally observed in vivo). The purpose of these experiments was to examine whether an induction of surface TF expression by CD14^+^-monocytes could be observed, which was found to be the case in response to almost all sera but at variable levels (Figure 4A), even though no direct relationships between ApoCIII serum concentrations and levels of surface TF induction were found (Figure 4B). Notably, the three patients displaying ApoCIII serum concentrations below 10 mg/dL and unable to promote TF induction (Figure 4B) did not present any acute thrombotic events, such as myocardial infarction or stroke, based on our records. 

As for ApoCIII, the effects of these sera on TF protein expression were accompanied by a concomitant induction of TF mRNA accumulation in the same cells (Supplementary Figure S3). More importantly, preincubation with specific ApoCIII-neutralizing rAbs (Figure 4C), but not isotype control antibodies reduced the ability of sera with measured concentrations of ApoCIII to upregulate TF surface protein (Figure 4C) and mRNA in CD14^+^-monocytes at variable levels but in a significant manner. By contrast, ApoCIII-neutralizing rAbs did not influence the capacity of LPS to upregulate TF protein expression (Figure 4C). On the other hand, ApoCIII-neutralizing rAbs were found to potently, but not completely, inhibit ApoCIII-induced TF expression (Figure 4C), likely because the amounts of antibodies used (60 µg/mL) were suboptimal to neutralize 50 µg/mL ApoCIII.

**Figure 5:**
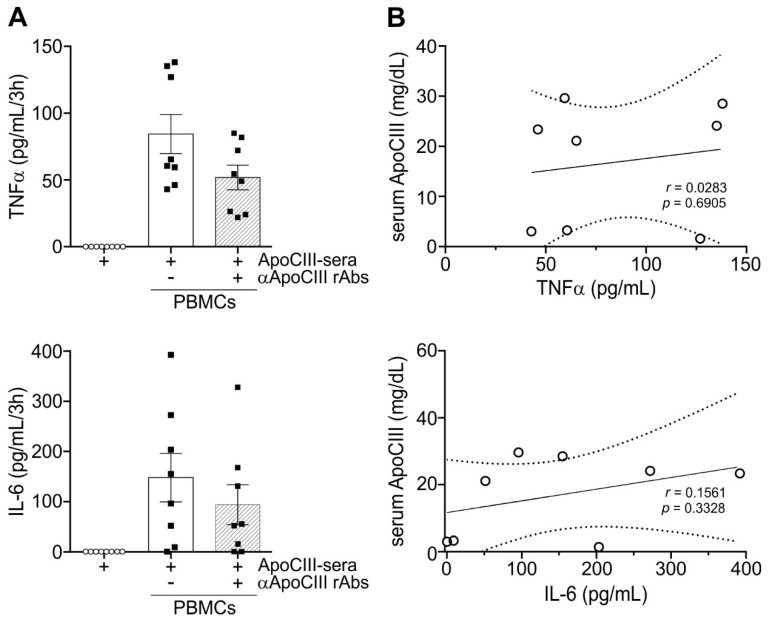
Effect of sera with measured concentrations of ApoCIII on TNFα and IL-6 release by PBMCs. Panel **A**: PBMCs isolated from buffy coats as reported in Section 4 were cultured for 3 h with the addition of ApoCIII-sera in the presence or absence of 60 µg/mL ApoCIII-neutralizing rAbs. After treatment, sera with known concentrations of ApoCIII (ApoCIII-sera) and PBMC-derived supernatants were collected and analyzed for TNFα and IL-6 content by ELISA. Data are expressed as mean± SEM (n = 8) and were analyzed by the Kruskal–Wallis test followed by Dunn’s multiple comparison test.. Panel **B** displays two scatter plots showing the correlation between TNFα or IL-6 release by PBMCs and the ApoCIII concentrations of the sera that were used as stimuli. The r-value determined by Pearson correlation is displayed together with the *p*-value.

As a further control, we also evaluated the ability of sera with measured concentrations of ApoCIII to promote the release of TNFα and IL-6 by PBMCs. As shown in Figure 5A, these sera did not contain any TNFα and IL-6 by themselves. However, once added to PBMCs, the sera samples triggered the release of variable but measurable amounts of both TNFα and IL-6 (Figure 5A). Again, no direct relationships between ApoCIII serum concentrations and the levels of TNFα and IL-6 release were found (Figure 5B). Finally, the release of TNFα and IL-6 promoted by those sera was also partially, but not completely, suppressed by ApoCIII-neutralizing rAbs (Figure 5A). 

Altogether, these data demonstrate that either ApoCIII alone or sera with measured concentrations of ApoCIII are able to promote the expression of surface TF in CD14^+^-monocytes. 

## 3. Discussion

In this study, we have demonstrated that human CD14^+^-monocytes, but not neutrophils, react in a “pro-inflammatory fashion” once exposed to ultra-purified apolipoprotein CIII. In fact, we have shown an increased expression of both pro-inflammatory cytokines (i.e., TNFα, IL-1β and IL-6) and TF mRNAs in ApoCIII-treated-CD14^+^-monocytes. ELISA experiments confirmed that ApoCIII-treated CD14^+^-monocytes release TNFα, IL-1β and IL-6 [7], while they were also found to rapidly display TF on their surface by flow cytometry. The latter results were reproduced using CD14^+^-monocytes incubated in a medium containing sera with measured concentrations of ApoCIII, an experimental condition probably closer to the physiological state of these cells in the bloodstream of cardiovascular patients. Finally, preincubation with specific ApoCIII-neutralizing rAbs variably but significantly reduced the ability of most sera containing measured concentrations of ApoCIII to upregulate TF protein in CD14^+^-monocytes, thus supporting the notion that ApoCIII may also stimulate CD14^+^-monocytes in vivo. Overall, our findings clearly demonstrate that ApoCIII, in addition to inducing a condition of “sterile inflammation” via IL-1β and other pro-inflammatory cytokine induction [7], activates CD14^+^-monocytes to express TF, the crucial initiator of extrinsic blood coagulation. 

We paid particular and continuous attention to obtain genuine results when using ApoCIII. In fact, in an extensive and careful preliminary work of verification, we found that almost all commercially available apolipoproteins, including ApoCIII, are—sometimes heavily—contaminated by endotoxin. Since ApoCIII triggers CD14^+^-monocyte responses via TLR4, the same receptor of LPS [7], and with LPS being one of the most potent activators of monocyte responses even at concentrations of picograms/mL, it was imperative to exclude false positive results determined by eventual contaminating endotoxin. For such a purpose, we purchased ApoCIII originally ultra-purified by the source company, which we further processed via an endotoxin removal kit to minimize as much as possible its endotoxin content. We also performed ad hoc experiments in which our working stimuli were pretreated with PMX, a drug that abolishes the effects of endotoxin [16]. All in all, our approach ultimately made sure that the effects of our ultra-purified ApoCIII on CD14^+^-monocytes were ”truly reliable”. In this regard, just for the implications for the inflammatory process, the setting up of "pure" uncontaminated experiments represents a crucial methodological point of our work.

Our findings are consistent with previous pieces of evidence suggesting a role of ApoCIII in exerting various biological effects on diverse immune cells, as well as in triggering and/or favoring the coagulative cascade [17]. For instance, it has been shown that ApoCIII induces endothelial activation [18] and, thus, the expression of vascular cell adhesion molecule-1 in endothelial cells, in turn increasing the adhesion of monocytic cells [4]. However, we would like to point out that it is not clear whether the ApoCIII used in these studies was endotoxin-free, as such information was missing. Thus, further work is necessary to confirm the reliability of the previously described observations. 

In the history of atherosclerosis research, monocytes have traditionally been viewed as key cells for both promoting early phases of plaque formation and favoring inflammation and inadequate repair of the damage [19]. Monocyte-rich plaques are also more vulnerable and prone to overimposed thrombosis [20]. In fact, inflammatory cytokines such as TNFα or interleukins strongly induce the expression of both full-length TF as well as alternatively spliced TF (asTF) in endothelial and blood cells [21,22]. Notably, among the different circulating blood cells, TF is exclusively expressed (under pathological conditions) by monocytes, which in turn release TF-positive microvesicles that bind to other cells, such as activated platelets, neutrophils and endothelial cells [23,24]. For these reasons, the role of TF activation in monocytes appears central in both triggering and amplifying the thrombotic process [23]. In this context, our group previously demonstrated that ApoCIII levels predict cardiovascular mortality in patients with severe coronary artery disease, and they are associated with enhanced plasma thrombin generation [11]. Elevated circulating levels of ApoCIII (but not other lipids or apolipoproteins) were also correlated with a progressive linear increase in factor II (FII) coagulant activity in the plasma of patients with or without coronary artery disease (CAD) [12]. The extent of this increase in subjects in the top quartile of ApoCIII concentration is comparable to that in heterozygous carriers of the FII G20210A mutation, a well-known genetic condition predisposing people to venous thrombosis. Consistently, in a prospective cohort study with long-term follow-up (12 years) aiming to investigate the association between plasma lipids and venous thromboembolic event (VTE) incidence in cardiovascular patients, subjects with high ApoCIII concentrations had an approximately three-fold increased risk of experiencing VTEs as compared with those with low ApoCIII levels [13]. ApoCIII was also demonstrated to be strongly associated with the activated FVII–antithrombin (FVIIa-AT) complex, which is an indirect marker of intravascular exposure of TF, thus providing a suggestion for ApoCIII-related activation of the extrinsic coagulation pathway [14]. In another recent study involving 127 patients with venous thromboembolism and 299 controls, concentrations of ApoCIII and -E were associated with several coagulation factors, including vitamin-K-dependent factors, as well as factor XI, factor VIII and von Willebrand factor levels [25]. All these clinical findings suggest an active interplay between ApoCIII-rich lipoproteins and activation of the coagulative process. The experimental data presented in this work seem to confirm this hypothesis by identifying a potential mechanism bridging circulating lipoproteins with inflammatory and atherothrombotic processes in a complex interaction with potentially harmful consequences. Upregulation of TF expression by monocytes may lead to both coagulation and inflammatory responses, thus rendering TF signaling an important target for anti-thrombotic as well as anti-inflammatory therapy [21,22]. By adding a further piece of evidence in understanding the mechanisms of TF expression in monocytes, which are key cells in the atherothrombotic process, our results provide further support to the rationale for an effective ApoCIII-lowering therapy in cardiovascular diseases. ApoCIII-specific anti-sense oligonucleotide therapy has been recently tested in humans, and based on our current findings, it should be interesting to verify its impact also on thrombotic risk. Similarly, the demonstrated anti-thrombotic properties of statins [24] may be consistently explained by their ApoCIII-lowering capacity.

Some limitations of our work also need to be acknowledged. In particular, we did not determine a precise molecular mechanism for ApoCIII TF induction, as our primary goal was to define whether TF expression could be induced by ApoCIII in monocytes. Additionally, as already mentioned, we believe that the explanation as to why ApoCIII-neutralizing rAbs did not completely inhibit ultrapure ApoCIII likely stands in the use of rAb amounts not sufficient to completely neutralize 50 µg/mL ApoCIII. However, mechanisms interfering with the Abs–antigen interaction/blocking (antibody dose, different epitope accessibility, interacting molecules, system complexity of the involved macromolecules and lipid particles, etc.) may also play a role. The variability of the response could also be due to the fact that ApoCIII can be associated to HDLs and VLDLs, and that these two lipoproteins can be variably distributed in an individual. In addition, ApoCIII is variously glycosylated, and three ApoCIII isoforms are recognized depending on the distribution of sialic acid residues [26]. It might be hypothesized that the different isoforms can make ApoCIII more or less sterically "invisible" to the ApoCIII-neutralizing rAbs. The proposed functions of sialylation include, in fact, stabilization of protein conformation, resistance to protease, charge and protein targeting [26]. Similarly, other circulating factors might (positively or negatively) modulate TF expression by ApoCIII-stimulated monocytes and also might be involved in modifying the interactions of ApoCIII with the specific neutralizing rAbs. It is known, for example, that ApoCIII is typically co-expressed with Apo E and ApoC-II but also with vitronectin and complement proteins (such as C2 and C1QC) [27]. Finally, it will be important to address whether or not ApoCIII triggers different signaling pathways to promote TNFα and IL-6 expression and surface TF.

Nevertheless, it was important to verify that the monocyte TF expression was ApoCIII-specific, not due (once again) to contamination or other unknown factors independent of ApoCIII. Finally, we are fully aware that more complex and in vivo studies are needed to definitively confirm the role of ApoCIII in infiltrating or atherosclerotic plaque residing monocytes/macrophages and neutrophils.

## 4. Materials and Methods

### 4.1. Materials

Ultrapure ApoCIII from human plasma was purchased from Academy Bio-Medical Company, Inc. (#33P-UP201, Houston, TX, USA), namely lots #110565, #10306 and #020377. The endotoxin content of the commercial ApoCIII was evaluated by LAL test (Pierce Chromogenic Endotoxin Quant Kit #A39552, Thermo Fisher Scientific, Waltham, MA, USA) in our lab and found to be 2.3 EU/mL for lot #110565, 2.53 EU/mL for lot #10306 and 6 EU/mL for lot #020377. High-capacity endotoxin removal spin columns purchased from Pierce-Thermo Fisher Scientific (#88273, Waltham, MA, USA) were then used to remove contaminating endotoxin from the ApoCIII preparations down to <0.07 EU/mL as an average. Recombinant human anti-ApoCIII antibodies (#HPAB-N0020-YC, clone 14C7, <1 EU/mg endotoxin) were purchased from Creative Biolabs (Shirley, NY, USA). Human IgG1 lambda, as a control for the 14C7 clone, was purchased from Southern Biotech (#0151L-14, Birmingham, AL, USA). ApoCIII concentrations in serum samples were measured using an automated turbidimetric immunoassay (Wako Pure Chemical Industries, Osaka, Japan), as previously described [11,12]. 

### 4.2. Cell Purification and Culture

Peripheral blood mononuclear cells (PBMCs) were isolated by density centrifugation of buffy coats from healthy donors (HDs) over Ficoll-Paque^TM^ PLUS (Cytiva, Uppsala, Sweden) and manipulated under endotoxin-free conditions [28]. Human CD14^+^-monocytes were isolated from PBMCs by anti-CD14 microbeads (Miltenyi Biotec, Bergisch Gladbach, Germany), reaching 98% purity [26]. Autologous neutrophils were isolated to approximately 99.7% purity after dextran sedimentation of granulocytes and hypotonic lysis of erythrocytes, followed by the removal of contaminating cells using the EasySep neutrophil enrichment kit (StemCell Technologies, Vancouver, Canada), as previously described [29]. Neutrophils and either autologous CD14^+^ monocytes or PBMCs (depending on the experiment) were suspended in RPMI 1640 medium (Corning Incorporated, Corning, NY, USA) supplemented with 10% low-endotoxin FBS (<0.5, from Sigma-Aldrich) at 5 × 10^6^/mL and 2.5×10^6^/mL, respectively, and then incubated with or without 50 µg/mL ApoCIII, 0.1 and 1 µg/mL LPS (ultrapure, *E. coli* 0111:B4 strain, from InvivoGen, San Diego, CA, USA) for CD14^+^-monocytes and neutrophils, respectively or 5 µM R848 (Resiquimod) (InvivoGen), an imidazoquinoline compound that activates immune cells via the TLR7/TLR8-dependent signaling pathway [30,31]. Cells were distributed in 24/96-well tissue culture plates (Corning Incorporated, Corning, NY, USA) and cultured at 37° in a 5 % CO_2_ atmosphere for up to 20 h. In some experiments, the stimuli were pretreated with 20 µg/mL polymyxin B sulphate (PMX, Sigma-Aldrich) before addition to the cells. In others, CD14^+^-monocytes or PBMCs were also incubated with 10% serum from 19 subjects enrolled within the cardiovascular cohort of the Verona Heart Study (VHS), having measurable ApoCIII serum levels (range 53–64 years, 79 % males) [11], in the presence or absence of 60 μg/mL anti-ApoCIII recombinant antibodies (rAbs) (or related human IgG1 lambda). After the desired incubation period, cells were processed for either total RNA preparation or FACS analysis.

### 4.3. RNA Purification and RT-Quantitative PCR (RT-qPCR) 

Total RNA was extracted with an RNeasy Mini Kit (Qiagen, Venlo, Limburg, The Netherlands) after cell lysis [30]. To completely remove any possible contaminating DNA, on-column DNase digestion with the RNase-free DNase set (Qiagen) was performed during total RNA isolation [30]. RNA integrity (RIN) was routinely found to be optimal (RIN ≥ 7.0). Purified RNA was reverse-transcribed into cDNA using the PrimeScript™ RT reagent Kit (Perfect Real Time) with random hexamer primers (Takara Bio Inc., Kusatsu, Japan), while qPCR was carried out using TB Green Premix Ex Taq (Tli RNaseH Plus) (Takara Bio Inc.). The sequences of the gene-specific primer pairs (Life Technologies) used in this study are as follows: *TNF*, forward GAGCACTGAAAGCATGATCC and reverse CGAGAAGATGATCTGACTGC; *IL1B*, forward AAACAGATGAAGTGCTCCTTCC and reverse AAGATGAAGGGAAAGAAGGTGC; *IL6*, forward CAAACAAATTCGGTACATCCTC and reverse CAAGTCTCCTCATTGAATCCA; *F3* (TF), forward CAGTGTTCAAGCAGTGATTCC and reverse ACATTCAGTGGGGAGTTCTC; *GAPDH*, forward AACAGCCTCAAGATCATCAGC and reverse GGATGATGTTCTGGAGAGCC. Data were calculated by Q-Gene software (http://www.gene-quantification.de/download.html, accessed on 20 January 2022) and are expressed as mean normalized expression (MNE) units after GAPDH normalization.

### 4.4. Flow Cytometry Analysis

For the analysis of TF surface expression, 2.5 × 10^5^ PBMCs were first incubated for 15 min on ice in 50 μL PBS containing 5 % human serum (to prevent nonspecific antibody binding) and then stained for 30 min on ice by fluorochrome-conjugated mAbs, namely PE anti-human TF/CD142 (Biolegend, NY2), Per-CP-Cy5.5 anti-human CD16 (Biolegend, 3G8), APC-Cy7 anti-human HLA-DR (Biolegend, L243), PE-VIO770 anti-human CD3 and CD19 (Miltenyi Biotec; REA613 and REA675, respectively) and APC anti-human CD14 (Miltenyi Biotec, REA599). Fluorochrome-conjugated antibodies were used at working dilutions as specified in the corresponding datasheets. Sample fluorescence was measured by a 16-color MACSQuant Analyzer (Miltenyi Biotec), while data analysis was performed on FlowJo software version 10.1 [32]. Cell viability was assessed by flow cytometry using Vybrant DyeCycleTM Violet (Thermo Fisher Scientific, Waltham, MA, USA), as previously described [30]. Phenotypic analysis under the various experimental conditions was performed on live cells, identified as Vybrant-negative cells. The fluorescence intensity relative to TF expression as well as the percentage of TF-positive cells were normalized to the fluorescence of the correspondent isotype control antibodies.

### 4.5. Detection of Extracellular Cytokines

Cytokine concentrations in cell-free supernatants were measured by commercial ELISA kits specific for human TNFα, IL-1β and IL-6 (Mabtech) according to the manufacturer’s instructions. The lowest detection limits of these ELISAs were 7.8 pg/mL for TNFα and IL-1β and 15.6 pg/mL for IL-6. An ELISA kit for human soluble tissue factor III/TF was purchased from R&D System, Bio-Techne, USA (lower detection limit of 7.8 pg/mL).

### 4.6. Statistical Analysis

Data are expressed as the mean ± SEM of the number of indicated experiments. Where applicable, normality distribution was estimated using the D’Agostino–Pearson, Shapiro–Wilk or Kolmogorov–Smirnov normality test. Statistical evaluation for normally distributed data was performed by unpaired *t*-test with Welch’s correction or one-way ANOVA followed by post hoc tests, depending on the type of experiments. Non-normally distributed data were assessed with the Mann–Whitney test or, for multiple group comparison, with the Kruskal–Wallis or Friedman test followed by multiple comparison tests. The tests used are indicated in the respective figure legends. Values of *p* < 0.05 were considered statistically significant. Statistical analysis was performed with GraphPad Prism v.7.0 software.

## Data Availability

This manuscript does not contain datasets.

## Supplementary Materials

The following supporting information can be downloaded at: https://www.mdpi.com/article/10.3390/ijms24032223/s1.
