# Supplementary material for: CD14+-Monocytes Exposed to Apolipoprotein CIII Express Tissue Factor
Source: Int J Mol Sci. 2023 Jan 22;24(3):2223. doi: 10.3390/ijms24032223 (PMC9916694; doi:10.3390/ijms24032223)
Supplement: Supplementary file 1 [file ijms-24-02223-s001.zip › ijms-1974361-SI.pdf]

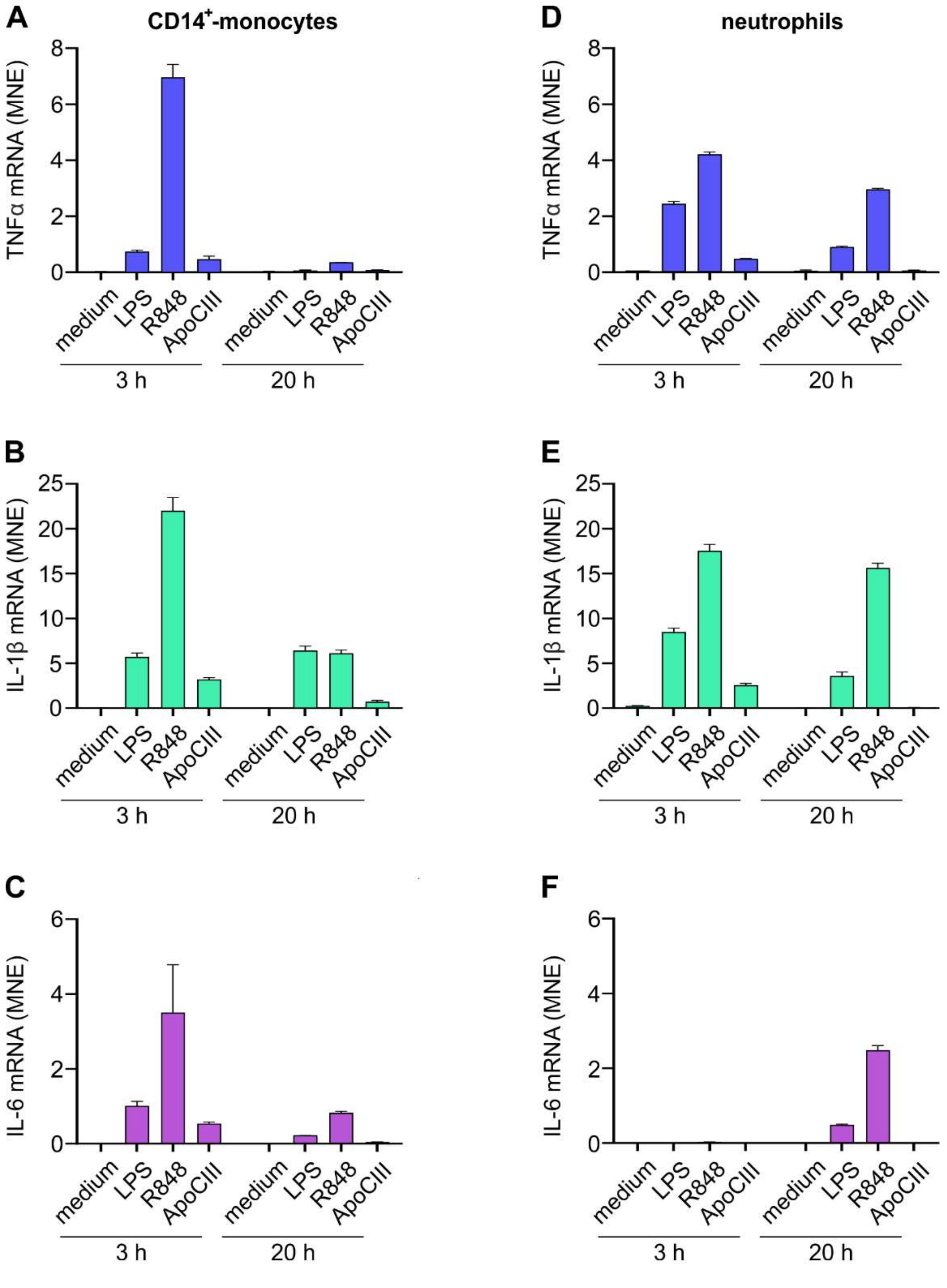

Figure S1. Kinetics of TNFα, IL-1β and IL-6 mRNA expression in human CD14+-monocytes and autologous neutrophils exposed to ApoCIII. Human CD14+-monocytes (**A, B, C**) and autologous neutrophils (**D, E, F**), purified as described in M&M Section 4.2, were incubated for 3 and 20 h in the absence (medium) or in the presence of 50 μg/mL ultrapure ApoCIII, 0.1 and 1 μg/mL LPS (for CD14+-monocytes and neutrophils, respectively) or 5 μM R848. Total RNA was then extracted and examined for TNFα (**A, D**), IL-1β (**B, E**) and IL-6 (**C, F**) mRNA by RT-qPCR. Gene expression is depicted as mean normalized expression (MNE) units after normalization to GAPDH mRNA of a representative experiment (out of 3 performed with similar results).

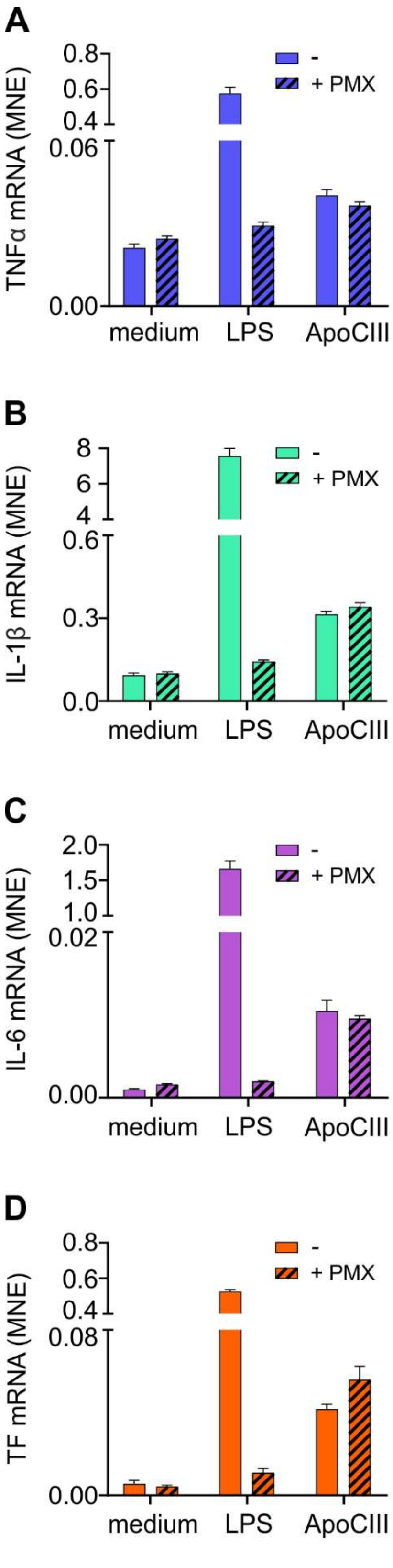

Figure S2. Effect of polymyxin B (PMX) on the ability of ApoCIII to induce TNFα, IL-1β, IL-6 and TF mRNA expression in human CD14+-monocytes. Human CD14+-monocytes were incubated for 3 h in the absence (medium) or the presence of 50 µg/mL ultrapure ApoCIII, 100 ng/mL LPS or 5 µM R848 previously pretreated with 20 µg/mL polymyxin B (PMX). Total RNA was then extracted and examined for TNFα (**A**), IL-1β (**B**) and IL-6 (**C**) and TF mRNA expression by qPCR. Gene expression is depicted as mean normalized expression (MNE) units after normalization to GAPDH mRNA of a representative experiment (out of 3 performed with similar results).

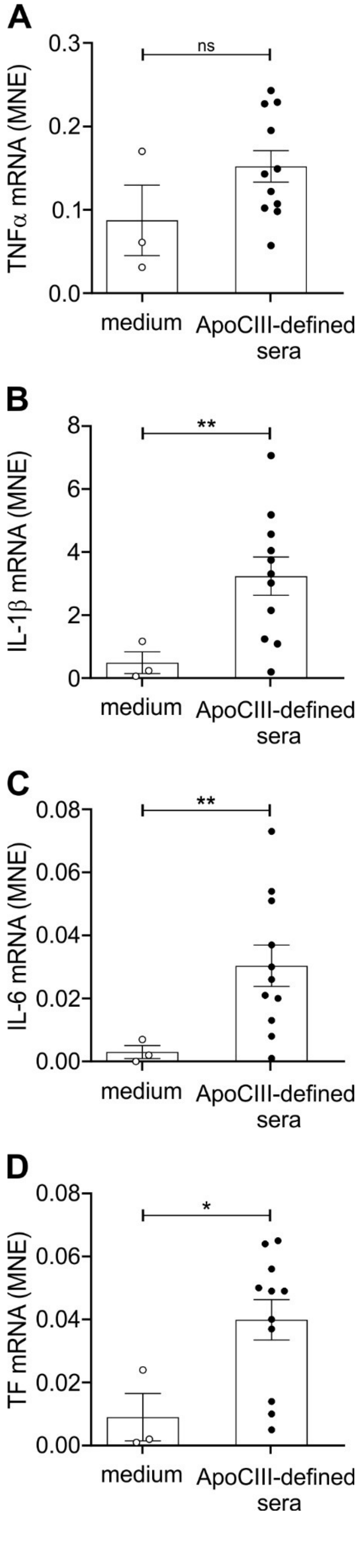

Figure S3. Expression of TNFα, IL-1β, IL-6 and TF mRNAs by human CD14⁺-monocytes exposed to ApoCIII-defined sera. Human CD14⁺-monocytes, purified as described in M&M, section 4.2, were incubated for 3 h in the absence (medium) or in the presence of 11 samples of ApoCIII-defined sera. Total RNA was then extracted and examined for TNFα (**A**), IL-1β (**B**), IL-6 (**C**) TF (**D**) mRNA expression by RT-qPCR. Gene expression is depicted as mean normalized expression (MNE) units after normalization to GAPDH mRNA (mean ± SEM, n = 3-11). Statistical analysis by unpaired t-test with Welch correction, ns = not significative, *p < 0.05, **p<0.01.
